# Screening for Hepatocellular Carcinoma Recurrence After Liver Transplantation: Prospective Validation of Binary Criteria

**DOI:** 10.1002/cam4.71428

**Published:** 2025-11-29

**Authors:** Wesley Dixon, Shaun Chandna, Jordan S. Sack, Meagan Gray, Christina N. Brown, Sampath Poreddy, Kai Ha, Michael R. Schoech, Stephen D. Zucker

**Affiliations:** ^1^ Division of Gastroenterology, Hepatology and Endoscopy Brigham & Women's Hospital Boston Massachusetts USA; ^2^ Division of Gastroenterology Olive View‐UCLA Medical Center Sylmar California USA; ^3^ Division of Gastroenterology and Hepatology The University of Alabama at Birmingham Birmingham Alabama USA; ^4^ Gastrointestinal Associates, Inc. Jenkintown Pennsylvania USA; ^5^ Gastroenterology Division Creighton University School of Medicine Omaha Nebraska USA; ^6^ Gastrohealth Cincinnati Ohio USA; ^7^ Division of Digestive Diseases University of Cincinnati Cincinnati Ohio USA

**Keywords:** cancer, cirrhosis, liver transplant, prediction, RETREAT, risk

## Abstract

**Background:**

Many centers screen all recipients for hepatocellular carcinoma (HCC) recurrence after liver transplantation (LT). This approach may add substantial burdens of cost, incidental findings, and, depending on the imaging modality, radiation and/or contrast‐induced complications. We created simple criteria to help clinicians identify low‐risk patients in whom post‐LT surveillance offers marginal utility.

**Methods:**

Criteria were developed retrospectively using adults with HCC who underwent LT at the University of Cincinnati from 2000 to 2014. Low‐risk patients had none of the following at LT: (1) outside Milan criteria, (2) alpha‐fetoprotein ≥ 200 ng/mL, (3) prior hepatectomy with vascular invasion or unknown histology, and (4) any vascular invasion or > 3 lesions on explant, or extrahepatic extension noted intraoperatively. Criteria were retrospectively validated in adults transplanted for HCC at Mass General Brigham from 2015 to 2023 and prospectively assessed at Cincinnati where low‐risk patients received no post‐LT surveillance.

**Results:**

Among 132 development cohort patients, 14 (10.6%) developed recurrent HCC at a median of 2.0 (range 0.1–9.7) years. Only 1 (1.1%) of the 91 (68.9%) patients deemed low‐risk by the proposed criteria developed recurrence compared to 13 (31.7%) of the high‐risk patients (*p* < 0.001). In the validation cohort (*n* = 188), recurrence occurred in 1 (0.9%) of 114 (60.6%) low‐risk and 16 (21.6%) high‐risk patients. In the prospective cohort (*n* = 55), none of 42 (76.4%) low‐risk patients developed recurrence versus 3 (23.1%) high‐risk patients (*p* = 0.011) after 8.0 (0.1–9.4) years of follow‐up. The criteria performed similarly to the RETREAT score 0 across cohorts (*n* = 375) in identifying patients without recurrence as low‐risk (negative predictive value 99.2% vs. 97.1%) but required screening significantly fewer patients (34.1% vs. 81.9%).

**Conclusions:**

These prospectively validated criteria offer clinicians a practical tool for easily and accurately determining which patients would benefit from surveillance for HCC recurrence after LT. Surveillance targeting only high‐risk patients could reduce the harms of excess screening.

**Abbreviations**
AFP: alpha‐fetoprotein
CT: computed tomography
DCP: des‐gamma‐carboxyprothrombin
HCC: hepatocellular carcinoma
LRT: locoregional therapy
LT: liver transplant
MELD: Model for End‐stage Liver Disease
MRI: magnetic resonance imaging
RETREAT: Risk Estimation of Tumor Recurrence After Transplant
RFA: radiofrequency ablation
TACE: transarterial chemoembolization
TARE: transarterial radioembolization

## 1 Introduction

Hepatocellular carcinoma (HCC) is the third leading cause of cancer death worldwide and occurs in the setting of cirrhosis in more than 80% of cases [1]. For select patients, liver transplantation (LT) is the preferred treatment for HCC as it eliminates the cancer, reduces recurrence risk by removing the diseased organ, and restores liver function [2]. However, studies estimate that HCC recurs in 8%–20% of patients, most often within three years after transplantation [3].

There is considerable variability in how transplant centers surveil patients for posttransplant HCC recurrence [4, 5]. Many centers perform serial cross‐sectional imaging and AFP measurement on all patients for up to five years regardless of individual risk for recurrence. This approach adds substantial burdens of cost, unintended consequences of incidental findings and, depending on the imaging modality, radiation and/or contrast‐induced complications. To personalize posttransplant screening through risk stratification, several models have been developed to estimate the likelihood of HCC recurrence using various combinations of histologic features (e.g., vascular invasion, degree of differentiation, tumor viability), serum markers (e.g., alpha‐fetoprotein (AFP), AFP‐L3, des‐gamma‐carboxyprothrombin (DCP)) and donor factors (e.g., age) [6, 7, 8, 9, 10, 11, 12, 13]. While these models can accurately characterize HCC recurrence risk across a continuum of risk, none have been designed to specifically identify the large number of patients who are at such low risk for recurrence that surveillance is unwarranted.

For instance, the Risk Estimation of Tumor Recurrence After Transplant (RETREAT) score is currently the most validated model used to guide posttransplant surveillance for HCC [13, 14, 15]. Current practice in some centers is to surveil posttransplant patients every six months for two years for RETREAT score 0–3, every six months for five years for RETREAT 4, and every three months for two years followed by every six months through five years for RETREAT ≥ 5. As patients with a RETREAT score of 0 have a relatively low risk of HCC recurrence (2.9% at 5 years), a few centers have elected to stop surveilling these individuals [13, 14, 15]. However, as only approximately 20% of those transplanted for HCC have a RETREAT score of 0, this strategy still necessitates surveillance of a sizeable majority of patients.

In the present study, we therefore aimed to develop a complementary, simple, binary categorization system designed to help clinicians identify a more substantial proportion of patients transplanted for HCC at very low risk of recurrence in whom post‐LT surveillance is of marginal utility. We first formulated these criteria using a retrospective cohort of patients at a single transplant center. We then validated them in a retrospective cohort of patients from an independent center over a nonoverlapping time frame. Finally, we prospectively confirmed the criteria performance in a cohort of liver transplant patients where those categorized as low risk did not undergo routine surveillance.

## 2 Methods

### 2.1 Development Cohort

We retrospectively identified adults (age ≥ 18 years) who underwent LT for HCC at the University of Cincinnati Medical Center between October 1, 2000 and June 30, 2014. Patients with prior LT or whose tumor histology demonstrated mixed features of HCC and cholangiocarcinoma were excluded. Operative details and liver histology from prior hepatic resections were obtained whenever possible. Duration of follow‐up was time from LT to death or most recent patient contact with the healthcare system. The number of tumors, size of the largest tumor, and the aggregate size of all tumors in the explant were considered regardless of viability. Tumor differentiation was graded based on the least differentiated feature of available lesions. The presence of vascular invasion was determined from the explant histology. Extrahepatic involvement was identified intraoperatively at the time of transplantation.

Standard post‐LT surveillance for the development cohort included contrast‐enhanced multiphasic abdominopelvic computed tomography (CT), non‐contrast chest CT, and serum AFP testing every three months for the first posttransplant year, followed by similar testing every six months until year five. In patients with impaired renal function, abdominal ultrasound or non‐contrast magnetic resonance imaging (MRI) scan was substituted for CT. Modification to this protocol was performed based on clinical circumstances, as determined by the responsible physician. The study was approved by the Institutional Review Board (IRB CR4‐2013‐4309).

### 2.2 Development of Risk‐Stratification Criteria for HCC Recurrence After Transplantation

We utilized inferential analysis to develop criteria that identified patients as low‐risk of HCC recurrence following LT. These criteria were created using parameters significantly associated with HCC recurrence in multivariable logistic regression analysis in the development cohort. Patients with incidental HCC were included in the analysis and classified in an identical manner to those with known pretransplant HCC. As our goal was to create criteria with very high negative predictive value while simultaneously minimizing the number needed to surveil, we experimented with adding additional parameters to the model that are well established to be predictive of recurrence but that occurred with low frequency in our cohort.

### 2.3 Validation Cohort

To validate the performance of our risk stratification criteria, we retrospectively identified adults with HCC who underwent LT at Mass General Brigham between January 1, 2015 and December 9, 2023. As with the development cohort, patients with prior LT or tumor histology demonstrating mixed features of HCC and cholangiocarcinoma were excluded. For the validation cohort, typical posttransplant surveillance included abdominopelvic CT, non‐contrast chest CT, and serum AFP testing every six months for the first two posttransplant years, then annually until year five. Abdominopelvic MRI could be substituted for CT based on physician preference. Data collection was approved by the Institutional Review Board (IRB 2022‐P000146).

### 2.4 Prospective Analysis of Risk‐Stratified HCC Surveillance

Commencing May 1, 2015, patients undergoing LT at the University of Cincinnati Medical Center for HCC characterized as low‐risk (meeting none of the proposed criteria, described below) received no posttransplant imaging surveillance for HCC recurrence. Patients identified as high‐risk (meeting any of the criteria) underwent HCC surveillance using a modified institutional protocol of multiphasic abdominopelvic CT, non‐contrast chest CT, and serum AFP measurement every six months for the first two posttransplant years and annually through year five. Ultrasound or MRI could be substituted for CT in those with impaired renal function. Modifications to this protocol could be considered as necessary by the responsible physician. Outcomes of patients with HCC who underwent LT between May 1, 2015 and April 30, 2017 (after implementation of this new surveillance stratification system), were analyzed through the period ending July 31, 2024. Approval for data collection and exemption from written consent requirements was provided by the Institutional Review Board (IRB CR4‐2013‐4309).

### 2.5 Statistical Analyses

Categorical variables were compared using chi‐squared or Fisher's exact tests. Continuous variables, reported using median and range, were compared using nonparametric Wilcoxon rank sum tests. Multivariable logistic regression was performed using parameters with correlations of *p* < 0.10 for model generation. Kaplan–Meier curves were analyzed by the log‐rank method. C‐statistics were calculated using time‐dependent receiver operating curve analysis. Criteria performance was compared with risk‐stratification by RETREAT score > 0 and RETREAT score > 2, thresholds that have been used in prior studies to identify high‐risk patients [13, 14, 16]. Hazard ratios (HR) with 95% confidence intervals (CI) for HCC recurrence were determined using Cox regression, with Fine–Gray analysis utilized to account for the competing event of death from non‐HCC causes. Statistical analyses were performed using R 4.2.1 (Vienna, Austria). Statistical significance was defined by two‐sided *p* < 0.05. All research was conducted in accordance with the Declarations of Helsinki and Istanbul.

## 3 Results

### 3.1 Development Cohort Characteristics and Correlates of HCC Recurrence

A total of 132 adults with HCC underwent LT at the University of Cincinnati Medical Center during the study period, 14 (10.6%) of whom developed recurrent HCC, with a median follow‐up of 3.3 (range: 0.0–13.0) years (Table 1). Serum AFP closest to transplantation was the only demographic or pretransplant parameter significantly associated with HCC recurrence (7.8 vs. 28.7 ng/mL; *p* = 0.023). There was no correlation between HCC recurrence and AFP on initial presentation for transplant evaluation whether considered as a continuous or dichotomous variable.

**TABLE 1:** Pretransplant, histopathologic, and posttransplant characteristics of adults with hepatocellular carcinoma who underwent liver transplant in the development cohort, stratified by tumor recurrence.

Characteristic	All patients, *n* = 132^a^	No recurrence, *n* = 118^a^	HCC recurrence, *n* = 14^a^	*p*
Age at LT (years)	57.2 (27.2–76.9)	57.3 (27.2–76.9)	53.6 (42.8–69.8)	0.299
Male	99 (75.0%)	87 (73.7%)	12 (85.7%)	0.516
White	110 (83.3%)	96 (81.4%)	14 (100%)	0.125
Follow‐up time (years)	3.3 (0.0–13.0)	3.3 (0.0–13.0)	2.9 (0.6–11.2)	0.997
Etiology of liver disease				
Hepatitis C	93 (70.5%)	81 (68.6%)	12 (85.7%)	0.230
Alcohol	26 (19.7%)	22 (18.6%)	4 (28.6%)	0.475
MASLD	15 (11.4%)	14 (11.9%)	1 (7.1%)	1.000
Cirrhosis	123 (93.2%)	109 (92.4%)	14 (100%)	0.596
Within Milan criteria				
At presentation	112 (84.8%)	101 (85.6%)	11 (78.6%)	0.445
At LT	129 (97.7%)	116 (98.3%)	13 (92.9%)	0.288
Prior hepatectomy	7 (5.3%)	6 (5.1%)	1 (7.1%)	0.553
Sorafenib use	12 (9.1%)	10 (8.5%)	2 (14.3%)	0.616
Any LRT	89 (67.4%)	81 (68.6%)	8 (57.1%)	0.383
RFA	51 (38.6%)	46 (39.0%)	5 (35.7%)	0.812
TACE	32 (24.2%)	29 (24.6%)	3 (21.4%)	1.000
TARE	13 (9.8%)	11 (9.3%)	2 (14.3%)	0.629
LRT procedures per patient	1.0 (0.0–5.0)	1.0 (0.0–5.0)	1.0 (0.0–2.0)	0.528
Time from LRT to LT (week)^b^	19.7 (0.9–115.1)	19.7 (0.9–115.1)	19.7 (7.0–55.1)	0.858
AFP prior to LT (ng/mL)	8.4 (1.2–1188)	7.8 (1.2–553.0)	28.7 (2.3–1188)	0.023
Number of HCC lesions	1.0 (0.0–12.0)	1.0 (0.0–12.0)	2.0 (0.0–7.0)	0.070
> 1 lesion	56 (42.4%)	47 (39.8%)	9 (64.3%)	0.080
> 2 lesions	29 (22.0%)	23 (19.5%)	6 (42.9%)	0.080
> 3 lesions	18 (13.6%)	13 (11.0%)	5 (35.7%)	0.025
Largest lesion size (cm)	2.7 (0.6–11.2)	2.6 (0.6–8.0)	3.5 (1.5–11.2)	0.015
Aggregate lesion size (cm)	3.4 (0.6–12.0)	3.1 (0.6–9.9)	5.1 (1.5–12.0)	0.016
Microvascular invasion	26 (19.7%)	18 (15.3%)	8 (57.1%)	< 0.001
Macrovascular invasion	5 (3.8%)	2 (1.7%)	3 (21.4%)	0.009
Tumor differentiation				
Poorly differentiated	10 (7.6%)	8 (6.8%)	2 (14.3%)	0.285
Moderately differentiated	62 (47.0%)	53 (44.9%)	9 (64.3%)	0.151
Well differentiated	51 (38.6%)	49 (41.5%)	2 (14.3%)	0.044
Unknown	9 (6.8%)	8 (7.6%)	1 (7.1%)	1.000
Incidental HCC	27 (20.5%)	24 (20.3%)	3 (21.4%)	1.000
Post‐LT AFP (ng/mL)	2.7 (0.5–1138)	2.7 (0.5–270.0)	6.2 (1.4–1138)	0.050
Immunosuppression				
Tacrolimus	111 (84.7%)	100 (85.5%)	11 (78.6%)	0.449
Mycophenolate mofetil	80 (61.1%)	73 (62.4%)	7 (50.0%)	0.369
Cyclosporine	13 (9.8%)	12 (10.3%)	1 (7.1%)	1.000
Steroids > 3 months after LT	88 (66.6%)	79 (66.9%)	9 (64.3%)	0.517
Acute cellular rejection	37 (28.0%)	34 (28.8%)	3 (21.4%)	0.756

Abbreviations: AFP, alpha‐fetoprotein; HCC, hepatocellular carcinoma; LRT, locoregional therapy; LT, liver transplant; MASLD, metabolic associated steatotic liver disease; RFA, radiofrequency ablation; TACE, transarterial chemoembolization; TARE, transarterial radioembolization.

^a^ Median (range); *n* (%).

^b^ From LRT closes to the time of LT.

Explant findings associated with HCC recurrence included: > 3 HCC lesions (35.7% vs. 11.0%; *p* = 0.025), largest HCC lesion size (3.5 vs. 2.6 cm; *p* = 0.015), aggregate lesion size (5.1 vs. 3.1 cm; *p* = 0.016), microvascular invasion (57.1% vs. 15.3%; *p* < 0.001), and macrovascular invasion (21.4% vs. 1.7%; *p* = 0.009). Conversely, well‐differentiated histology was less common in those patients with recurrence (14.3% vs. 41.5%; *p* = 0.044). Extrahepatic spread was unexpectedly encountered intraoperatively at the time of transplantation in two recipients, both of whom developed HCC recurrence despite attempted wide excision. Incidental diagnosis of HCC on explant was equally common among patients who did and did not develop recurrence (21.4% vs. 20.3%; *p* = NS). Although the median posttransplant AFP was within the normal range, recipients with recurrent HCC were found to have modestly higher AFP than those without recurrence (6.2 vs. 2.7 ng/mL; *p* = 0.050).

### 3.2 Outcomes of Patients With HCC Recurrence

**FIGURE 1:**
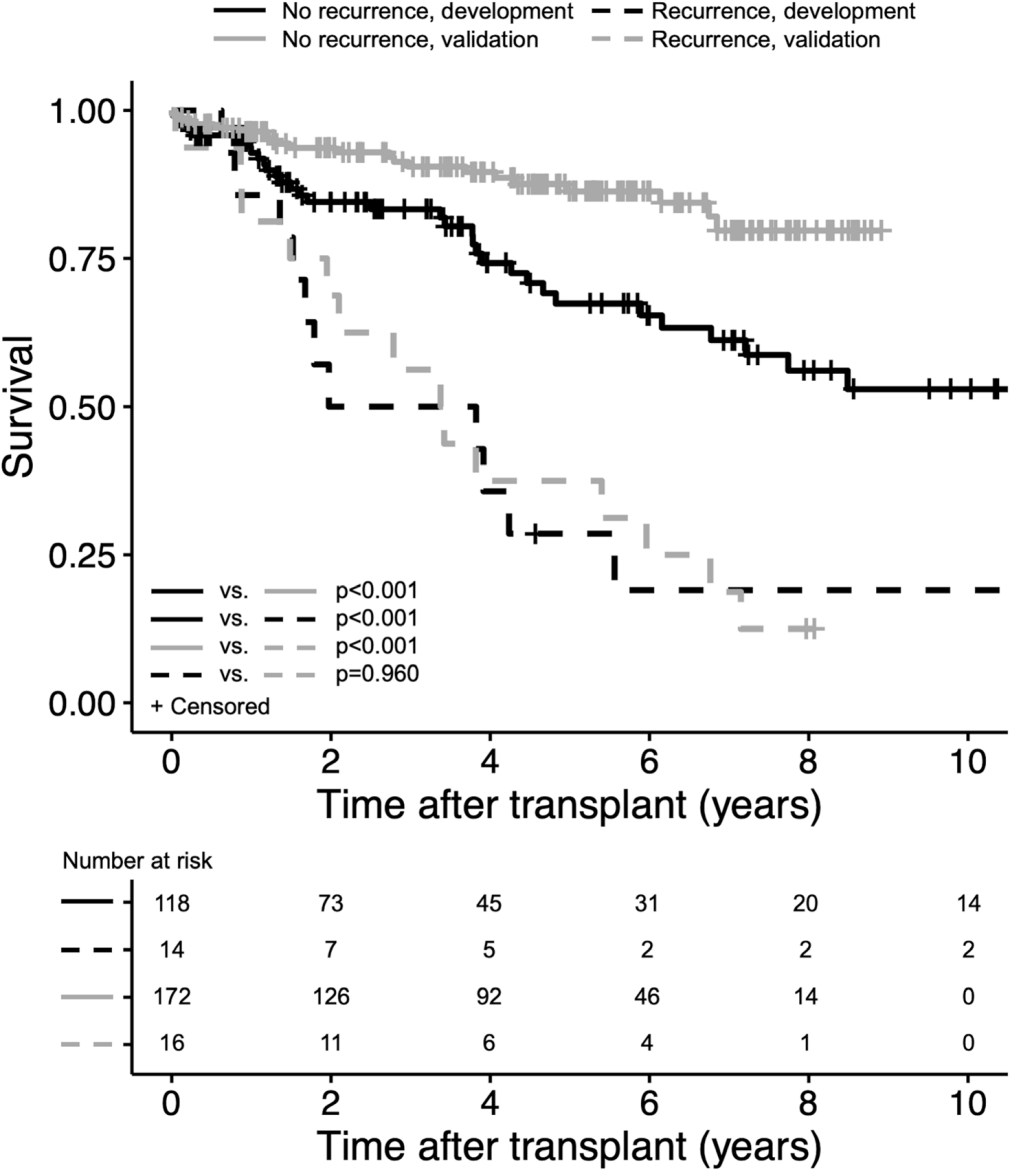
Survival for liver transplant recipients with or without recurrent hepatocellular carcinoma in the development and validation cohorts.

Of the 14 patients in the development cohort who experienced HCC recurrence, median time to recurrence was 2.0 (range: 0.1–9.7) years (Figure S1). Ten (71.4%) recurrences occurred within three years after LT and 13 (92.9%) occurred within five years. The most common site of recurrence was within the liver or liver bed (71.4%), followed by lung (28.6%), bone (21.4%), and adrenals (14.3%), with 21.4% of patients presenting with multi‐site involvement. Patients with recurrent HCC exhibited significantly worse survival than those who did not recur (*p* < 0.001) (Figure 1). Survival after recurrence did not correlate with frequency of surveillance imaging or AFP measurement.

### 3.3 Development of the Binary Categorization Model for Patients With HCC Undergoing LT

Multiple logistic regression analysis incorporating variables with *p* < 0.10 by univariable analysis identified two factors that remained significantly associated with HCC recurrence: extrahepatic spread or any vascular invasion (micro‐ and/or macrovascular) on explant histology (OR 6.1 [95% CI 1.1–34.0], *p* = 0.038) and most recent serum AFP prior to LT (OR 1.00 per 1 ng/mL [95% CI 1.00–1.01], *p* = 0.044). To facilitate identification of a dichotomous pretransplant AFP threshold, we compared the performance of AFP cutoff values of 25, 50, 100, 200, and 500 ng/mL, for significance. An AFP threshold of 200 ng/mL was found to be the strongest discriminator for identifying patients with HCC recurrence (2.4% vs. 30.0%; *p* < 0.001).

As we aimed to develop criteria with high negative predictive value while minimizing the number needed to surveil, we experimented with adding additional parameters well‐established to be predictive of HCC recurrence but that occurred with sufficiently low frequency in our cohort. These ultimately included the patient being outside of Milan criteria, having > 3 lesions on explant, intraoperative tumor extension, or having undergone a prior hepatectomy with evidence of any vascular invasion (or unknown histology). Taken together, patients who met none of the following criteria were categorized as being at low‐risk for HCC recurrence after LT:

1. Outside Milan criteria at the time of LT (after downstaging following LRT), or
2. AFP ≥ 200 ng/mL at the time of LT (after downstaging following LRT), or
3. Prior hepatectomy for HCC with evidence of any vascular invasion (or unknown histology), or
4. Any vascular invasion or > 3 lesions (regardless of viability) in the liver explant, or extrahepatic extension noted intraoperatively.

**FIGURE 2:**
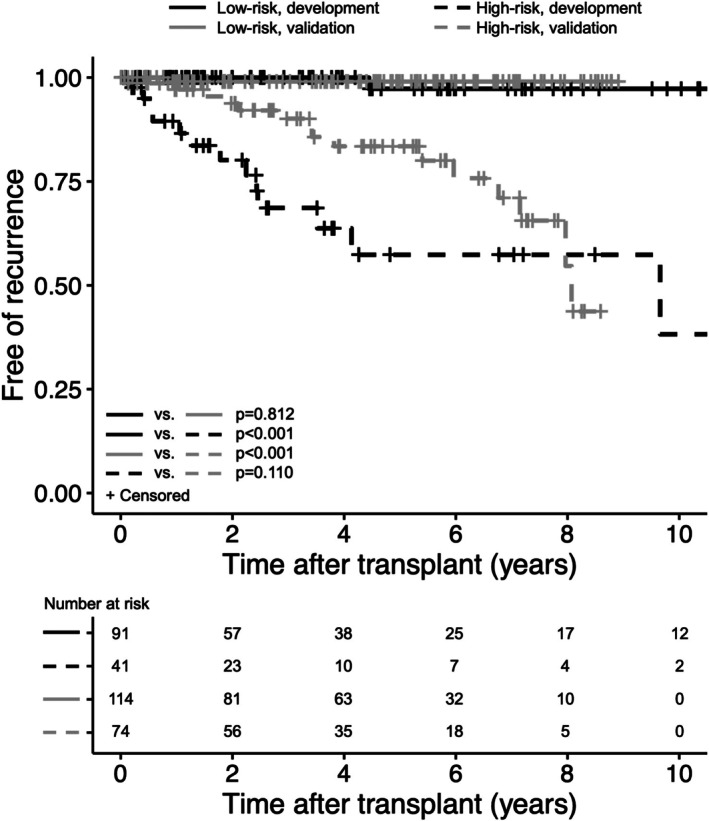
Proportion of liver transplant recipients free of hepatocellular carcinoma recurrence in the development and validation cohorts. Patients are stratified into low‐ and high‐risk groups based on proposed risk‐stratification criteria.

When applied to the development cohort, 91 (68.9%) patients met none of the criteria and were categorized as low‐risk, whereas the 41 (31.1%) patients who met at least one criterion were classified as high‐risk (Table S1). Only 1 (1.1%) patient who was classified as low‐risk developed osseous HCC recurrence 4.4 years after LT (Figure 2). Of high‐risk patients, 13 (31.7%) developed recurrent HCC over the entire study period. This translates to a sensitivity of 92.9%, specificity of 76.3%, positive predictive value of 31.7%, and negative predictive value of 98.9%. By the proposed criteria, the number of high‐risk patients needed to surveil to detect one HCC recurrence was 3.1. In comparison, 123 (93.2%) patients stratified as high‐risk using a threshold RETREAT score > 0, resulting in a sensitivity of 92.9%, specificity of 6.8%, and number needed to screen 9.5. Using a threshold of RETREAT score > 2, 37 (28.0%) patients were classified as high‐risk (sensitivity 71.4%, specificity 77.1%, number needed to screen 3.7), with 4.2% of patients in the low‐risk cohort developing HCC.

### 3.4 Validation Cohort Characteristics and Risk Stratification

To validate criteria performance, we retrospectively identified 188 patients at Mass General Brigham who underwent LT for HCC between January 1, 2015 and December 9, 2023 (Table 2). Of these, 16 (8.5%) developed recurrent HCC after LT over a median follow‐up of 4.3 (0.0–8.9) years. Time from LT to diagnosis of recurrent HCC in the validation cohort was similar to the development cohort (1.6 vs. 2.0 years, respectively; *p* = 0.759). Key differences in the validation cohort compared to the development cohort included less pre‐LT sorafenib utilization (0% vs. 9.1%; *p* < 0.001), a higher proportion of patients who received any LRT (93.6% vs. 67.4%; *p* < 0.001), and more LRT procedures per patient (2.0 vs. 1.0; *p* < 0.001). The development and validation cohorts were otherwise similar with regard to demographics.

**TABLE 2:** Pretransplant, histopathologic, and posttransplant characteristics of adults with hepatocellular carcinoma who underwent liver transplantation in the validation cohort, stratified by hepatocellular carcinoma recurrence.

Characteristic	All patients, *n* = 188^a^	No recurrence, *n* = 172^a^	HCC recurrence, *n* = 16^a^	*p*
Age at LT (years)	62.0 (24.0–73.0)	62.0 (24.0–73.0)	63.5 (54.0–71.0)	0.915
Male	155 (82.5%)	142 (82.6%)	13 (81.3%)	1.000
White	146 (77.7%)	132 (76.7%)	14 (87.5%)	0.530
Follow‐up time (years)	4.3 (0.0–8.9)	4.3 (0.0–8.9)	3.4 (0.05–8.1)	0.614
Etiology of liver disease				
Hepatitis C	96 (51.1%)	87 (50.6%)	9 (56.3%)	0.664
Alcohol	61 (32.5%)	56 (32.6%)	5 (31.3%)	0.915
MASLD	32 (17.0%)	30 (17.4%)	2 (12.5%)	1.000
Cirrhosis	183 (97.3%)	167 (97.1%)	16 (100%)	1.000
Within Milan at LT	177 (94.2%)	167 (97.1%)	10 (62.5%)	< 0.001
Prior hepatectomy	11 (5.9%)	11 (6.4%)	0 (0%)	0.603
Sorafenib use	0 (0%)	0 (0%)	0 (0%)	1.000
Any LRT	176 (93.6%)	161 (93.6%)	15 (93.8%)	1.000
RFA	124 (66.0%)	115 (66.9%)	9 (56.3%)	0.392
TACE	66 (35.1%)	57 (33.1%)	9 (56.3%)	0.064
TARE	20 (10.6%)	19 (11.1%)	1 (6.3%)	1.000
LRT procedures per patient	2.0 (0.0–7.0)	1.0 (0.0–7.0)	2.0 (1.0–4.0)	0.589
Time from LRT to LT (week)^b^	41.4 (0.6–165.0)	42.7 (0.6–165.0)	29.4 (3.3–85.9)	0.331
AFP prior to LT (ng/mL)	5.2 (1.1–17,718)	5.0 (1.1–603)	30.2 (1.8–17,718)	0.023
Number of HCC lesions	2.0 (1.0–15.0)	2.0 (1.0–15.0)	2.0 (1.0–12.0)	0.020
> 1 lesion	105 (55.9%)	95 (55.6%)	10 (62.5%)	0.592
> 2 lesions	66 (35.1%)	59 (34.5%)	7 (43.8%)	0.459
> 3 lesions	45 (23.9%)	39 (22.8%)	6 (37.5%)	0.222
Largest lesion size (cm)	3.2 (0.1–9.5)	3.1 (0.1–9.5)	3.4 (0.9–6.2)	0.247
Aggregate lesion size (cm)	4.4 (0.1–27.5)	4.3 (0.1–27.5)	5.7 (1.7–22.6)	0.938
Microvascular invasion	20 (10.6%)	16 (9.3%)	4 (25.0%)	0.073
Macrovascular invasion	8 (4.3%)	5 (2.9%)	3 (18.8%)	0.018
Tumor differentiation				
Poorly differentiated	9 (4.8%)	8 (4.7%)	1 (6.2%)	0.559
Moderately differentiated	98 (52.1%)	88 (51.2%)	10 (62.5%)	0.385
Well differentiated	25 (13.3%)	23 (13.4%)	2 (12.5%)	1.000
Unknown	56 (29.8%)	53 (30.8%)	3 (18.8%)	0.401
Incidental HCC	12 (6.4%)	10 (5.8%)	2 (12.5%)	0.271
Post‐LT AFP (ng/mL)	3.0 (0.8–9625)	3.0 (0.8–900.4)	3.9 (1.8–9625)	0.177
Steroids > 3 months after LT	134 (71.3%)	124 (72.1%)	10 (62.5%)	0.401
Acute cellular rejection	29 (15.4%)	25 (14.5%)	4 (25.0%)	0.279

Abbreviations: AFP, alpha‐fetoprotein; HCC, hepatocellular carcinoma; LRT, locoregional therapy; LT, liver transplant; MASLD, metabolic associated steatotic liver disease; RFA, radiofrequency ablation; TACE, transarterial chemoembolization; TARE, transarterial radioembolization.

^a^ Median (range); *n* (%).

^b^ From LRT closest to the time of LT.

HCC recurred most commonly in the lung or bone (44.1% each) followed by the liver or liver bed (25.0%). Recurrence was detected as a result of patient‐reported symptoms in 50.0%, routine surveillance imaging in 37.8%, and rising AFP in 12.2%. Similar to the development cohort, survival in the validation cohort was worse in patients who developed recurrence (*p* < 0.001) (Figure S1). There was no difference in the survival of the patients who developed HCC recurrence between the cohorts (*p* = 0.960). Notably, in patients without recurrence, overall survival was higher in the validation compared to the development cohort (*p* < 0.001), which may reflect the earlier time frame of the development cohort. This contention is supported by the identical survival between the validation and prospective cohort (see below), which were studied over concordant time frames.

Within the validation cohort, 114 (60.6%) patients were classified as low‐risk by the proposed criteria, only 1 (0.9%) of whom developed recurrent HCC at 0.5 years after LT (Figure 2). A total of 74 (39.4%) patients were classified as high‐risk, 15 (20.3%) of whom developed recurrence (Table S2). Performance characteristics of the criteria in the validation cohort were similar to those in the development cohort, with sensitivity 93.8%, specificity 65.7%, positive predictive value 20.3%, and negative predictive value 99.1%. In comparison, 141 patients (75%) were considered high‐risk when stratified by RETREAT score > 0, corresponding to a sensitivity of 93.8% and specificity of 26.7% for HCC recurrence. Using RETREAT score > 2, 39 (20.7%) patients were identified as high‐risk with a sensitivity of 43.8% and specificity 81.4%. A total of 9 (6.0%) low‐risk patients by RETREAT > 2 developed HCC recurrence. By our criteria, the number of high‐risk patients needed to surveil to detect an HCC recurrence was 4.9, compared to 9.4 for RETREAT > 0, and 5.6 for RETREAT > 2.

### 3.5 Prospective Analysis of Risk‐Stratified Surveillance for HCC Recurrence

**FIGURE 3:**
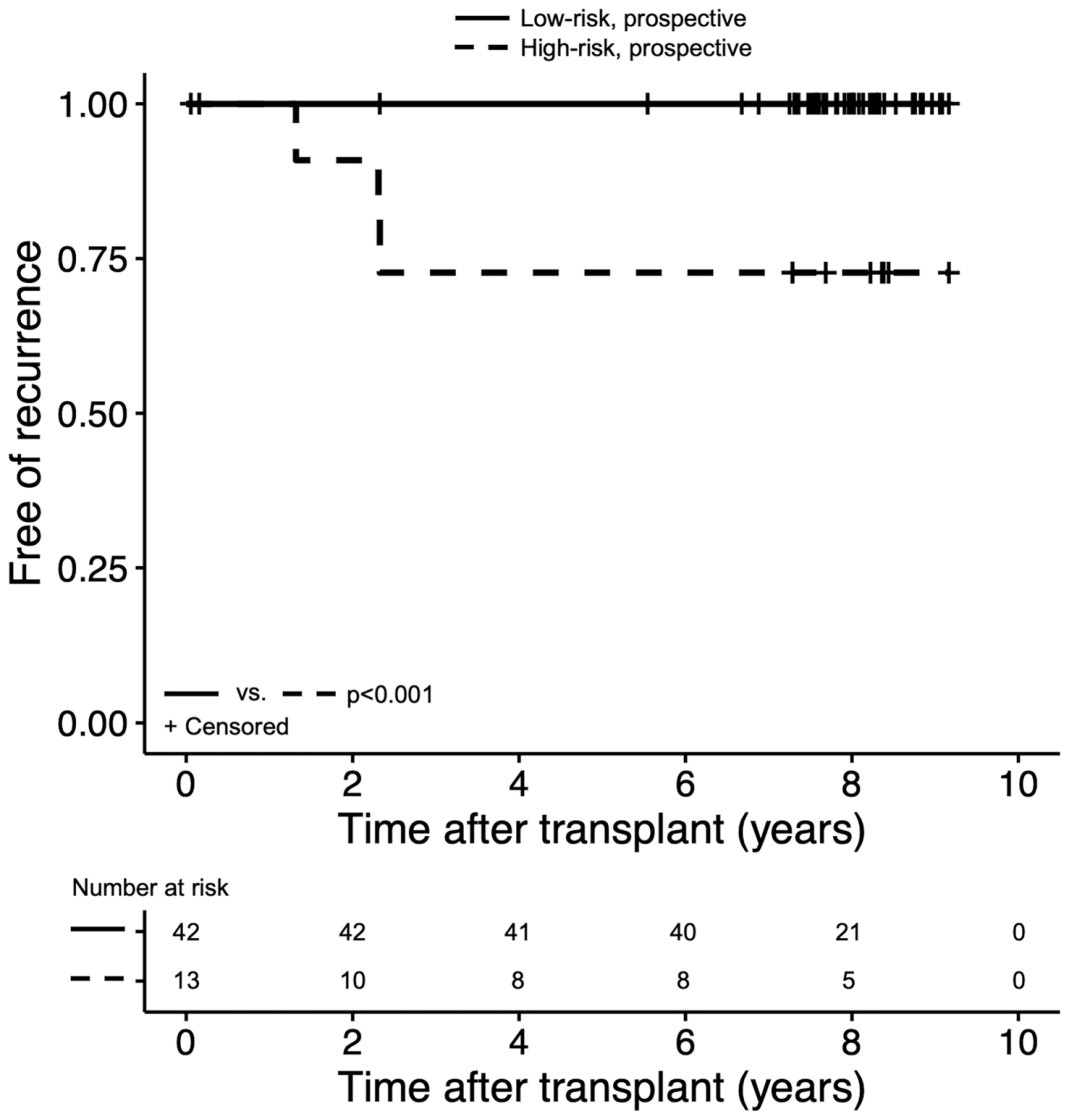
Proportion liver transplant recipients free of hepatocellular carcinoma recurrence in the prospective cohort. Patients are stratified into low‐ and high‐risk groups based on novel risk‐stratification criteria.

In light of the excellent performance characteristics of the novel criteria, from May 2015 through April 2017, patients undergoing LT for HCC at the University of Cincinnati Medical Center who were characterized as low‐risk by the proposed criteria received no posttransplant imaging surveillance for HCC recurrence. During this inclusion period, 55 patients with HCC underwent LT (Table 3). Over a substantial median follow‐up of 8.0 (2.3–9.2) years, 42 (76.4%) patients were categorized as low‐risk, none of whom developed recurrent HCC. Three (23.1%) of 13 patients stratified as high‐risk developed recurrence, corresponding to 5.5% of the total prospective cohort (Figure 3). Recurrence occurred in the bone (66.7%), liver or liver bed (33.3%), lung (33.3%), and in multiple sites (66.7%) at a median 1.1 (1.0–1.4) years. All three patients who developed recurrence died within two years of diagnosis (Figure S2). Criteria performance in the prospective cohort was sensitivity 100%, specificity 80.8%, positive predictive value 23.1%, and negative predictive value 100%. The number needed to surveil to detect one HCC recurrence was 4.3. In comparison, 43 patients (78.2%) were considered high‐risk by RETREAT > 0, resulting in a sensitivity of 100%, specificity 23.1%, and number needed to surveil 14.3. Thirteen (23.6%) patients were defined as high‐risk by RETREAT > 2, resulting in a sensitivity of 100%, specificity 80.8%, and number needed to surveil 4.3. Of note, while 13 patients were categorized as high‐risk by both the proposed criteria and RETREAT > 2, the groups were not identical. Survival was similar to the validation cohort among those who developed recurrence (*p* = 0.131) and those who did not (*p* = 0.610).

**TABLE 3:** Pretransplant, histopathologic, and posttransplant characteristics of adults with hepatocellular carcinoma who underwent liver transplant in the prospective cohort, stratified into low‐ and high‐risk by the proposed binary criteria.

Characteristic	All patients, *n* = 55^a^	Low‐risk, *n* = 42^a^	High‐risk, *n* = 13^a^	*p*
Age at LT (years)	62.2 (45.5–76.4)	63.7 (45.5–76.4)	60.4 (51.5–68.2)	0.293
Male	39 (70.9%)	28 (66.7%)	11 (84.6%)	0.304
White	48 (87.3%)	37 (88.1%)	11 (84.6%)	0.664
Follow‐up time (years)	8.0 (0.1–9.2)	8.0 (2.3–9.2)	7.3 (0.1–9.2)	0.076
Etiology of liver disease				
Hepatitis C	29 (52.8%)	21 (50.0%)	8 (61.5%)	0.467
Alcohol	18 (32.7%)	12 (28.6%)	6 (46.1%)	0.314
MASLD	13 (23.6%)	9 (21.4%)	4 (30.8%)	0.480
Cirrhosis	53 (96.4%)	40 (95.2%)	13 (100%)	1.000
Within Milan at LT	55 (100%)	42 (100%)	13 (100%)	1.000
Prior hepatectomy	3 (5.5%)	1 (2.4%)	2 (15.4%)	0.136
Sorafenib use	15 (27.3%)	11 (26.2%)	4 (30.8%)	0.734
Any LRT	43 (78.2%)	35 (83.3%)	8 (61.5%)	0.129
RFA	1 (1.8%)	1 (2.4%)	0 (0%)	1.000
TACE	31 (56.4%)	24 (57.1%)	7 (53.9%)	0.834
TARE	24 (43.6%)	19 (45.2%)	5 (38.5%)	0.667
LRT procedures per patient	1.0 (0.0–8.0)	1.0 (0.0–5.0)	1.0 (0.0–8.0)	0.719
Time from LRT to LT (week)^b^	20.1 (0.6–160.8)	20.7 (0.6–160.8)	8.4 (1.0–93.3)	0.089
AFP prior to LT (ng/mL)	7.3 (1.1–894.6)	7.0 (1.1–159.4)	10.0 (2.1–894.6)	0.163
Number of HCC lesions	1.0 (0.0–9.0)	1.0 (0.0–3.0)	4.0 (1.0–9.0)	0.004
> 1 lesion	23 (41.8%)	15 (35.7%)	8 (61.5%)	0.099
> 2 lesions	13 (23.6%)	5 (11.9%)	8 (61.5%)	< 0.001
> 3 lesions	7 (12.7%)	0 (0%)	7 (53.9%)	< 0.001
Largest lesion size (cm)	2.6 (0.9–12.0)	2.5 (0.9–7.0)	3.1 (1.6–12.0)	< 0.001
Aggregate lesion size (cm)	3.1 (0.0–21.1)	3.0 (0.0–13.5)	6.5 (1.6–21.1)	0.001
Microvascular invasion	6 (10.9%)	0 (0%)	6 (46.2%)	< 0.001
Macrovascular invasion	1 (1.8%)	0 (0%)	1 (7.7%)	0.236
Tumor differentiation				
Poorly differentiated	2 (4.8%)	1 (3.5%)	1 (7.7%)	0.528
Moderately differentiated	31 (73.8%)	22 (75.9%)	9 (69.2%)	0.713
Well differentiated	9 (21.4%)	6 (20.7%)	3 (23.1%)	1.000
Unknown	13 (23.6%)	13 (31.0%)	0 (0%)	< 0.001
Incidental HCC	8 (14.6%)	5 (11.9%)	3 (23.1%)	0.376
Post‐LT AFP (ng/mL)	2.7 (1.0–13.8)	2.6 (1.1–13.8)	2.7 (1.0–8.8)	0.459
Steroids > 3 months after LT	1 (1.9%)	1 (2.4%)	0 (0%)	1.000
Acute cellular rejection	5 (9.1%)	5 (11.9%)	0 (0%)	1.000
HCC recurrence	3 (5.5%)	0 (0%)	3 (23.1%)	0.011

*Note:* Low‐risk patients did not receive surveillance for tumor recurrence after transplant.

Abbreviations: AFP, alpha‐fetoprotein; HCC, hepatocellular carcinoma; LRT, locoregional therapy; LT, liver transplant; MASLD, metabolic associated steatotic liver disease; RFA, radiofrequency ablation; TACE, transarterial chemoembolization; TARE, transarterial radioembolization.

^a^ Median (range); *n* (%).

^b^ From LRT closest to the time of LT.

### 3.6 Aggregated Cohort Data

**FIGURE 4:**
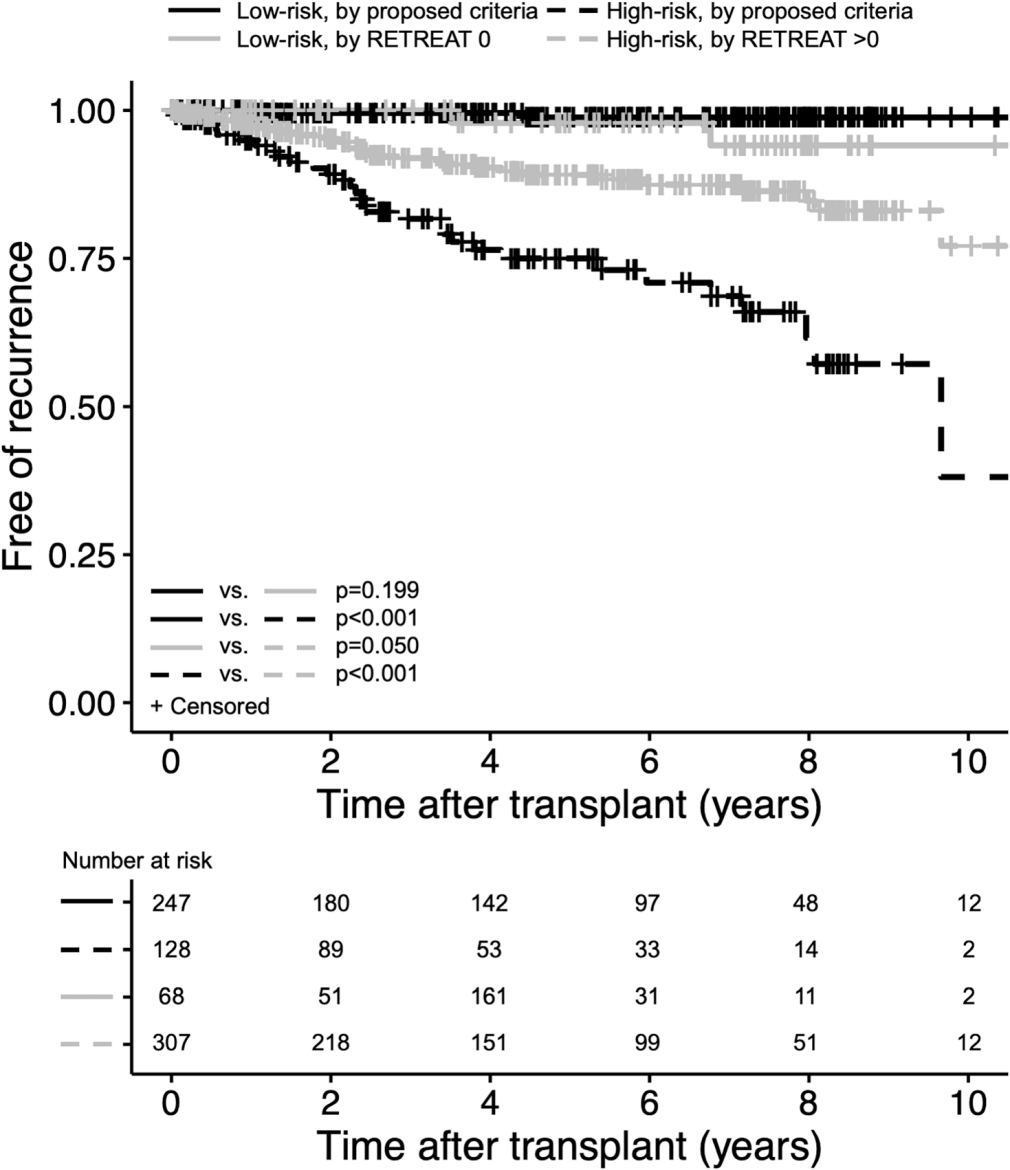
Proportion of liver transplant recipients with hepatocellular carcinoma recurrence after aggregating the development, validation, and prospective cohorts (*n* = 375). Patients are stratified into low‐ and high‐risk groups based on either the proposed binary criteria or RETREAT score > 0.

Synthesized data for the individual and aggregated cohorts are shown in Table 4. Across the pooled retrospective and prospective cohorts (*n* = 375), the cumulative sensitivity and specificity of our novel criteria were 93.9% and 71.6%, respectively, versus 93.9% and 19.3% for RETREAT > 0 (Figure 4) and 60.6% and 79.8% for RETREAT > 2 (Figure S3). The negative predictive value for our criteria, RETREAT > 0, and RETREAT > 2 was 99.2%, 97.1%, and 95.5%, respectively. Hence, the novel criteria performed similarly to RETREAT > 0 in identifying patients without recurrent HCC as low‐risk, but required screening a substantially lower proportion of the cohort (34.1% vs. 81.9%, respectively). Conversely, HCC recurred in a higher proportion of patients characterized as low‐risk by RETREAT > 2 (4.5%) as compared with our criteria (0.8%). C‐statistics from time‐dependent receiver operating curve analysis for HCC recurrence by our criteria at five and ten years were 0.82 and 0.91, respectively, compared to 0.59 and 0.54 for RETREAT > 0 and 0.77 and 0.75 for RETREAT > 2 (Figure S4). As determined by Cox regression, the cause‐specific hazard ratio (HR) for HCC recurrence in patients categorized as high‐risk by our criteria compared to those at low‐risk was 36.6 (95% CI 8.76–153.3, *p* < 0.001), while the risk of death from non‐HCC causes did not differ between risk groups (HR 1.2, 95% CI 0.7–2.1, *p* = 0.42). The highly similar subdistribution HR determined by Fine–Gray analysis (HR 35.1, 95% CI 8.41–146.0, *p* < 0.001) supports that the competing risk of death from non‐HCC causes did not bias the results.

**TABLE 4:** Summarized results of the proposed criteria across the cohorts.

Characteristic	Cohort			
	Development^a^	Validation^a^	Prospective^a^	Aggregated^a^
Number of patients	132	188	55	375
Follow‐up time (years)	3.3 (0.0–13.0)	4.3 (0.0–8.9)	8.0 (2.3–9.2)	4.3 (0.0–13.0)
Recurrences	14 (10.6%)	16 (8.5%)	3 (5.5%)	33 (8.8%)
High‐risk patients	41 (31.1%)	74 (39.4%)	13 (23.6%)	128 (34.1%)
Recurrence	13 (31.7%)	15 (20.3%)	3 (23.1%)	31 (24.2%)
No recurrence	28 (68.3%)	59 (79.7%)	10 (76.9%)	97 (75.8%)
Low‐risk patients	91 (68.9%)	114 (60.6%)	42 (76.4%)	247 (65.9%)
Recurrence	1 (1.1%)	1 (0.9%)	0 (0%)	2 (0.8%)
No recurrence	90 (98.9%)	113 (99.1%)	42 (100%)	245 (99.2%)
Sensitivity	92.9%	93.8%	100%	93.9%
Specificity	76.3%	65.7%	80.8%	71.6%
Positive predictive value	31.7%	20.3%	23.1%	24.2%
Negative predictive value	98.9%	99.1%	100%	99.2%

^a^ Median (range); *n* (%).

## 4 Discussion

Despite widespread use of pretransplant LRT, AFP threshold limits, and longer wait times from listing to transplantation for MELD exception, there remains a nontrivial risk of HCC recurrence after LT. Rates of posttransplant HCC recurrence in our development and validation cohorts were 10.6% and 8.5%, respectively, which mirror ranges reported in the literature [3]. For this reason, the American Association for the Study of Liver Diseases recommends surveillance of patients transplanted for HCC with regular contrast‐enhanced multiphasic CT or MRI abdomen and CT chest scans [17]. However, as posttransplant recurrence rates for HCC are relatively low, it would be optimal to focus surveillance on patients at heightened risk [18].

Prior models have been created to stratify patients according to HCC recurrence risk after LT in order to guide the frequency of posttransplant surveillance [15]. However, none of these existing models were designed to specifically identify patients at such low risk for recurrence that surveillance is unwarranted. Hence, our objective was to devise a complementary, simple, and practical binary method for identifying a larger proportion of LT recipients at such low risk for HCC recurrence that they can reasonably forego posttransplant surveillance. A successful set of criteria would ensure high negative predictive value while minimizing the number of patients flagged for screening.

We validated a set of binary criteria to achieve this aim. The proposed criteria performed similarly well in retrospective analyses of patients from two independent centers (despite no temporal overlap) and in a separate prospective cohort. When data from 375 patients in all cohorts were combined, approximately two‐thirds met none of the criteria and had a risk of HCC recurrence below 1%. In contrast, the remaining one‐third who met at least one criterion manifested a 20%–25% likelihood of developing recurrence, with a hazard ratio of 36 compared to low‐risk individuals. Assuming a median of two years to HCC recurrence and that all recurrences are diagnosed within five years, if one were to apply the surveillance protocol from the development cohort, we calculate that 25 imaging studies would be required to detect one recurrence in high‐risk patients compared with 738 imaging studies needed to detect one recurrence in low‐risk patients. While a few transplant programs forego screening in patients with a RETREAT score of 0, our proposed criteria exhibited similar sensitivity (93.9% vs. 93.9%) but superior specificity (71.6% vs. 19.3%), translating to substantially fewer patients requiring screening (34.1% vs. 81.9%, respectively). One could therefore envision a two‐stage screening algorithm in which patients who are high‐risk by our proposed criteria proceed to validated RETREAT‐based stratification.

This represents one of the first prospective studies of a surveillance protocol in which liver recipients transplanted for HCC classified as being at low risk for recurrence did not undergo routine HCC surveillance after transplantation. Notably, in our prospective cohort, none of the patients categorized as low risk developed HCC recurrence after a median posttransplant follow‐up of eight years. The only other prospective study of recurrent HCC, which also included a subset of patients who did not undergo surveillance, reported a much shorter median follow‐up (2.2 years) [15]. Given that the overwhelming majority of recurrences occur within five years of transplant (at which point most programs cease surveillance), the substantial follow‐up time achieved by our study is a key strength and minimizes the potential for lead time bias in the patients not receiving routine surveillance imaging. Our findings demonstrate the feasibility of employing a surveillance protocol for HCC recurrence that spares low‐risk patients, which constitute a majority of those transplanted for HCC, the potential costs and risks associated with unnecessary imaging. The binary classification of individuals as high‐ or low risk affords clinicians a practical tool for easily and accurately determining which posttransplant patients would benefit from surveillance.

A limitation of the present study is the relatively small sample size of the cohorts. However, it is reassuring that the demographics and recurrence rates were similar across the development, validation, and prospective cohorts, and that the combined 375 patients were risk‐stratified by the criteria with excellent predictive accuracy. The broad time frame over which the cohorts were derived similarly attests to the generalizability of our findings. Another limitation is the possibility that an HCC recurrence could have been overlooked as a result of insufficient follow‐up time, or that recurrences were missed in prospective cohort patients classified as low‐risk due to nonuse of imaging surveillance. Reassuringly, the long duration of follow‐up (median 8.0 years in the prospective cohort) makes this highly unlikely. In addition, Fine–Gray analyses support that the competing risk of death from non‐HCC causes is unlikely to have biased our findings. It is important to point out that only a minority (37%) of recurrences in the validation cohort were detected by surveillance imaging, supporting that recurrent HCC is identified in most patients when symptoms and/or altered biochemistries prompt further diagnostic evaluation. Moreover, whether posttransplant surveillance permits identification of recurrent HCC at an early enough stage to positively impact outcomes remains unproven [19]. Indeed, we did not observe any improvement in survival following HCC recurrence among the cohorts, despite substantially different time frames. Further validation of the proposed criteria in a large, modern, multicenter cohort would be highly informative.

The results of this study do not apply to all patients. Patients whose tumor histology demonstrated mixed features of HCC and cholangiocarcinoma were notably excluded. Patients in our study did not receive immunotherapy, as this was not available nor commonly utilized over most of the cohort time frames. Promising biomarkers AFP‐L3 and DCP were also not available over much of the time the data were collected and thus were not investigated. Future studies investigating risk‐stratified recurrence screening approaches may be able to incorporate these variables. Inclusion of other promising monitoring methods, such as circulating tumor DNA, may also improve patient outcomes [20].

In conclusion, we have shown that the proposed set of criteria is a simple and practical tool to help identify which patients do or do not require routine surveillance imaging for HCC recurrence after transplant. Implementation of this approach could help transplant clinicians minimize the pursuit of incidental findings, reduce costs, prevent potential complications, and lessen inconvenience for patients. Indeed, the center at which the prospective study was conducted continues to employ this binary approach in clinical practice.

## Author Contributions

**Wesley Dixon:** conceptualization, writing – original draft, methodology, visualization, writing – review and editing, formal analysis, software, data curation, project administration. **Shaun Chandna:** conceptualization, methodology, writing – review and editing, formal analysis, data curation. **Jordan S. Sack:** conceptualization, writing – review and editing, formal analysis, supervision. **Meagan Gray:** writing – review and editing, methodology. **Christina N. Brown:** methodology, writing – review and editing. **Sampath Poreddy:** methodology, writing – review and editing. **Kai Ha:** methodology, writing – review and editing. **Michael R. Schoech:** methodology, writing – review and editing. **Stephen D. Zucker:** conceptualization, writing – review and editing, methodology, formal analysis, supervision, data curation.

## Funding

The authors have nothing to report.

## Ethics Statement

This study was approved by the University of Cincinnati Institutional Review Board (IRB CR4‐2013‐4309) as well as the Mass General Brigham Institutional Review Board (IRB 2022‐P000146).

## Conflicts of Interest

The authors declare no conflicts of interest.

## Supporting information


**Appendix S1:** cam471428‐sup‐0001‐Supinfo.docx.

## Data Availability

The data that support the findings of this study are available from the corresponding author upon reasonable request.
