# Supplementary material for: Screening for Hepatocellular Carcinoma Recurrence After Liver Transplantation: Prospective Validation of Binary Criteria
Source: Cancer Med. 2025 Nov 29;14(23):e71428. doi: 10.1002/cam4.71428 (PMC12664103; doi:10.1002/cam4.71428)
Supplement: Supplementary file 1 — Appendix S1: cam471428‐sup‐0001‐Supinfo.docx. [file CAM4-14-e71428-s001.docx]

**Supplemental Figure 1:** Time from liver transplantation to diagnosis of hepatocellular carcinoma (HCC) recurrence in the development and validation cohorts


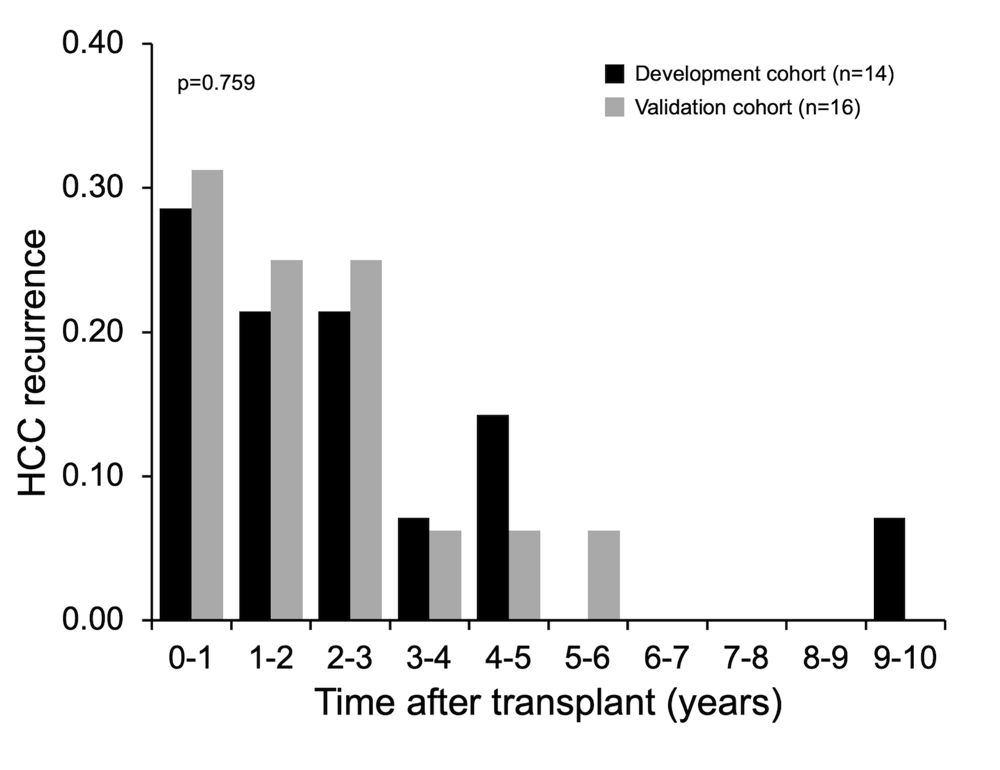


**Supplemental Figure 2:** Survival curves for liver transplant recipients with or without recurrent hepatocellular carcinoma in the prospective cohort.


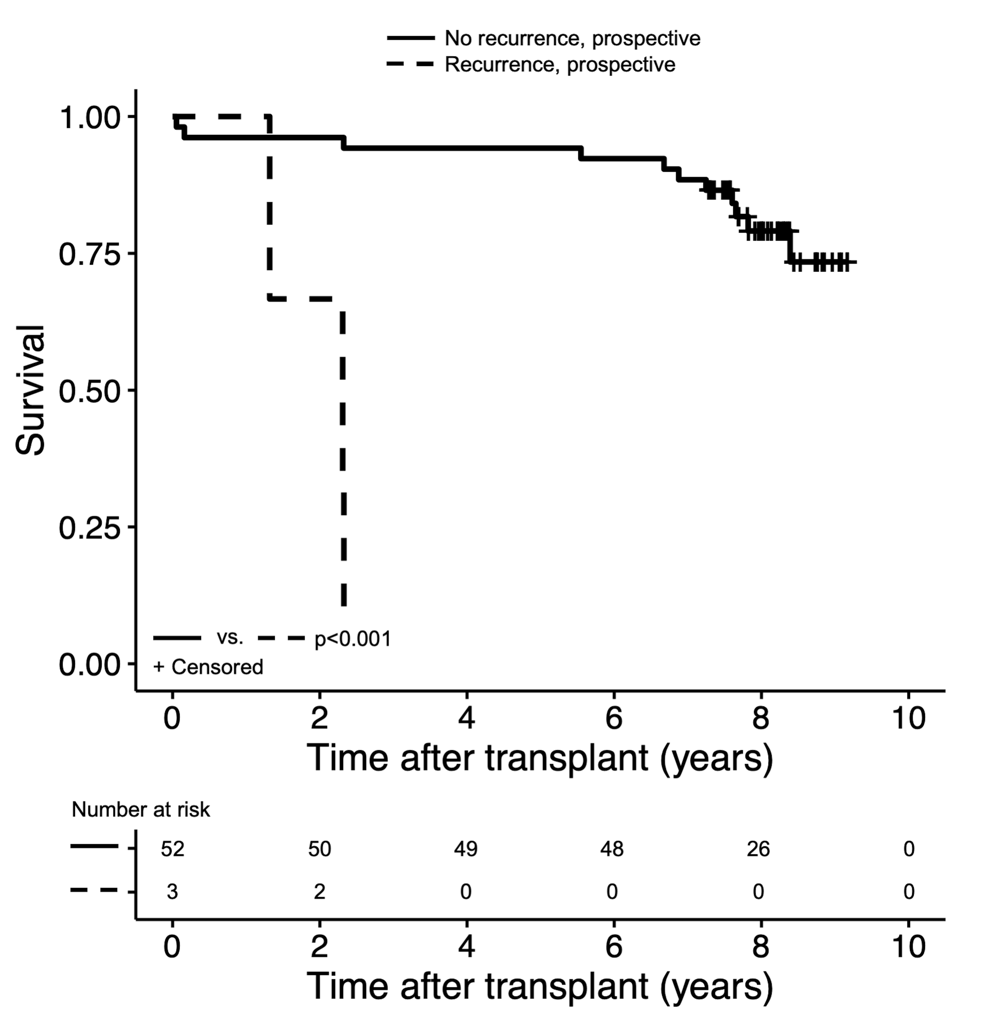
**Supplemental** **Figure 3:** Proportion of liver transplant recipients with hepatocellular carcinoma recurrence after aggregating the development (n=132), validation (n=188), and prospective cohorts (n=55) (total n=375). Patients are stratified into low- and high-risk groups based on either the proposed binary criteria or RETREAT score >2.


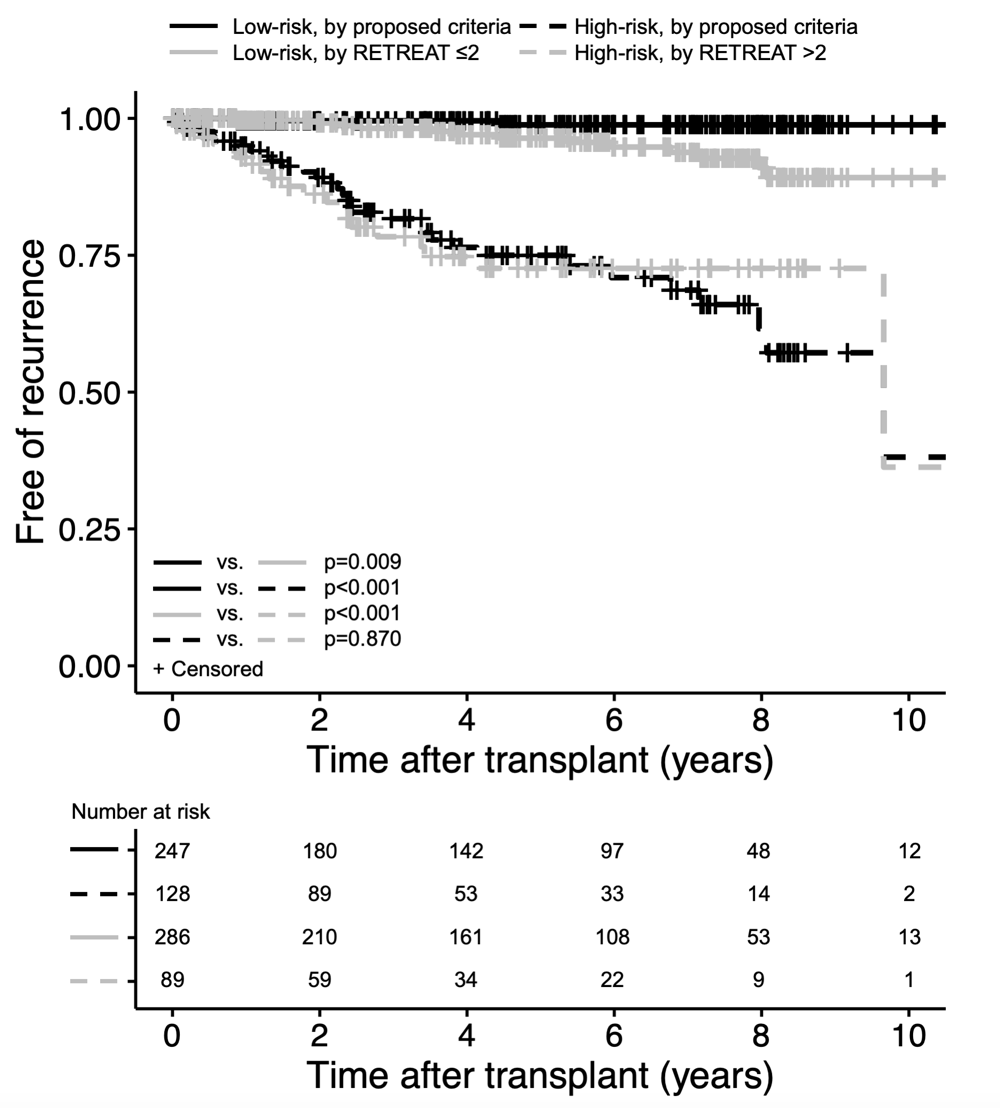
**Supplemental Figure 4:** C-statistic values from time-dependent receiver operating curve analysis comparing the proposed criteria, RETREAT score >0, and RETREAT score >2 in predicting risk of hepatocellular carcinoma recurrence in the pooled retrospective and prospective cohorts (n=375).


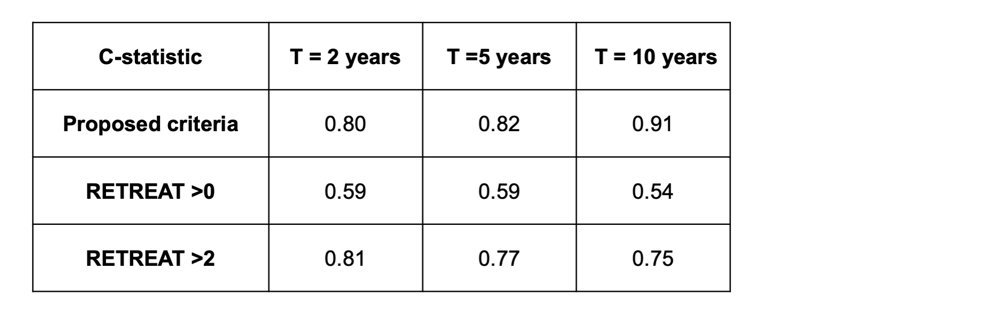
**Supplemental Table 1:** Pre-transplant, histopathologic, and post-transplant characteristics of adults with hepatocellular carcinoma who underwent liver transplant in the development cohort, stratified into low- and high-risk by the proposed criteria.

| **Characteristic** | **All patients**,  n = 132*^1^* | **Low-risk**,  n = 91*^1^* | **High-risk**,  n = 41*^1^* | **p-value** |
| --- | --- | --- | --- | --- |
| Age at LT (y) | 57.2 (27.2-76.9) | 57.3 (35.4-76.5) | 57.0 (27.2-76.9) | 0.690 |
| Male | 99 (75.0%) | 65 (71.4%) | 34 (82.9%) | 0.158 |
| White | 110 (83.3%) | 74 (81.3%) | 36 (87.8%) | 0.355 |
| Follow-up time (y) | 3.3 (0.0-13.0) | 3.4 (0.0-13.0) | 2.4 (0.2-11.0) | 0.225 |
| Etiology of liver disease |  |  |  |  |
| Hepatitis C | 93 (70.5%) | 66 (72.5%) | 27 (65.8%) | 0.437 |
| Alcohol | 26 (19.7%) | 18 (19.8%) | 8 (19.5%) | 0.971 |
| MASLD | 15 (11.4%) | 8 (8.8%) | 7 (17.1%) | 0.234 |
| Cirrhosis | 123 (93.2%) | 86 (94.5%) | 37 (90.2%) | 0.458 |
| Within Milan criteria |  |  |  |  |
| At presentation | 112 (84.8%) | 80 (87.9%) | 32 (78.1%) | 0.144 |
| At LT | 129 (97.7%) | 91 (100%) | 39 (92.7%) | 0.028 |
| Prior hepatectomy | 7 (5.3%) | 2 (2.2%) | 5 (12.2%) | 0.030 |
| Sorafenib use | 12 (9.1%) | 6 (6.6%) | 6 (14.6%) | 0.189 |
| Any LRT | 89 (67.4%) | 62 (68.1%) | 27 (65.9%) | 0.796 |
| RFA | 51 (38.6%) | 38 (41.8%) | 13 (31.7%) | 0.272 |
| TACE | 32 (24.2%) | 18 (19.8%) | 14 (34.1%) | 0.075 |
| TARE | 13 (9.8%) | 9 (9.9%) | 4 (9.8%) | 1.00 |
| LRT procedures per patient | 1.0 (0.0-5.0) | 1.0 (0.0-5.0) | 1.0 (0.0-5.0) | 0.837 |
| Time from LRT to LT (wk)^2^ | 19.7 (0.9-115.1) | 21.0 (0.9-115) | 16.9 (0.9-82.9) | 0.202 |
| AFP (ng/mL) | 8.4 (1.2-1,188) | 8.0 (1.2-161) | 9.5 (2.2-1,188) | 0.108 |
| Number of HCC lesions | 1.0 (0.0-12.0) | 1.0 (1.0-3.0) | 3.0 (0.0-12.0) | <0.001 |
| >1 lesion | 56 (42.4%) | 27 (29.7%) | 29 (70.7%) | <0.001 |
| >2 lesions | 29 (22.0%) | 7 (7.7%) | 22 (53.7%) | <0.001 |
| >3 lesions | 18 (13.6%) | 0 (0%) | 18 (43.9%) | <0.001 |
| Largest lesion size (cm) | 2.7 (0.6-11.2) | 2.5 (0.6-7.5) | 3.0 (1.0-11.2) | 0.163 |
| Aggregate lesion size (cm) | 3.4 (0.6-12.0) | 2.9 (0.6-9.5) | 4.5 (0.6-12.0) | <0.001 |
| Microvascular invasion | 26 (19.7%) | 0 (0%) | 26 (63.4%) | <0.001 |
| Macrovascular invasion | 5 (3.8%) | 0 (0%) | 5 (12.2%) | 0.002 |
| Tumor differentiation |  |  |  |  |
| Poorly differentiated | 10 (7.6%) | 5 (5.5%) | 5 (12.2%) | 0.292 |
| Moderately differentiated | 62 (47.0%) | 36 (40%) | 26 (63.4%) | 0.025 |
| Well differentiated | 51 (38.6%) | 42 (46%) | 9 (22.0%) | 0.003 |
| Unknown | 9 (6.8%) | 8 (8.8%) | 1 (2.4%) | 0.273 |
| Incidental HCC | 27 (20.5%) | 20 (22.0%) | 7 (17.1%) | 0.518 |
| Post-LT AFP (ng/mL) | 2.7 (0.5-1,138) | 2.7 (0.5-270.0) | 3.0 (0.8-1,138) | 0.291 |
| Immunosuppression |  |  |  |  |
| Tacrolimus | 111 (84.7%) | 72 (79.1%) | 39 (95.1%) | 0.026 |
| Mycophenolate mofetil | 80 (61.1%) | 56 (61.5%) | 24 (58.6%) | 0.688 |
| Cyclosporine | 13 (9.8%) | 12 (13.2%) | 1 (2.4%) | 0.062 |
| Steroids >3 months after LT | 88 (66.7%) | 57 (62.6%) | 31 (75.6%) | 0.101 |
| Acute cellular rejection | 37 (28.0%) | 26 (28.6%) | 11 (26.8%) | 0.837 |
| HCC recurrence | 14 (10.6%) | 1 (1.1%) | 13 (31.7%) | <0.001 |
| *^1^* Median (range); n (%). ^2^ From LRT closest to the time of LT | | | | |
| *AFP, alpha-fetoprotein; HCC, hepatocellular carcinoma; LRT, locoregional therapy; LT, liver transplant; MASLD, metabolic associated steatotic liver disease; RFA, radiofrequency ablation; TACE, transarterial chemoembolization; TARE, transarterial radioembolization.* | | | | |

**Supplemental** **Table 2:** Pre-transplant, histopathologic, and post-transplant characteristics of adults with hepatocellular carcinoma who underwent liver transplantation in the validation cohort, stratified into low- and high-risk by the proposed criteria.

| **Characteristic** | **All patients**,  n = 188*^1^* | **Low-risk**,  n = 114*^1^* | **High-risk**,  n = 74*^1^* | **p-value** |
| --- | --- | --- | --- | --- |
| Age at LT (y) | 62.0 (24.0-73.0) | 62.0 (26.0-73.0) | 62.5 (24.0-71.0) | 0.931 |
| Male | 155 (82.5%) | 91 (79.8%) | 64 (86.5%) | 0.241 |
| White | 146 (77.7%) | 89 (78.1%) | 57 (77.0%) | 0.867 |
| Follow-up time (y) | 4.3 (0.0-8.9) | 4.3 (0.1-8.9) | 3.9 (0.0-8.6) | 0.592 |
| Etiology of liver disease |  |  |  |  |
| Hepatitis C | 96 (51.1%) | 61 (53.5%) | 35 (47.3%) | 0.405 |
| Alcohol | 61 (32.5%) | 33 (29.0%) | 28 (37.8%) | 0.203 |
| MASLD | 32 (17.0%) | 23 (20.2%) | 9 (12.2%) | 0.153 |
| Cirrhosis | 183 (97.3%) | 111 (97.4%) | 72 (97.3%) | 1.00 |
| Within Milan at LT | 177 (94.2%) | 114 (100%) | 63 (85.1%) | <0.001 |
| Prior hepatectomy | 11 (5.9%) | 1 (0.9%) | 10 (13.5%) | <0.001 |
| Sorafenib use | 0 (0%) | 0 (0%) | 0 (0%) | 1.00 |
| Any LRT | 176 (93.6%) | 104 (91.2%) | 72 (97.3%) | 0.130 |
| RFA | 124 (66.0%) | 74 (64.9%) | 50 (67.6%) | 0.707 |
| TACE | 66 (35.1%) | 37 (32.5%) | 29 (39.2%) | 0.345 |
| TARE | 20 (10.6%) | 12 (10.5%) | 8 (10.8%) | 0.951 |
| LRT procedures per patient | 2.0 (0.0-7.0) | 1.0 (0.0-5.0) | 2.0 (0.0-7.0) | <0.001 |
| Time from LRT to LT (wk)^2^ | 41.4 (0.6-165.0) | 47.9 (2.0-165.0) | 34.8 (0.6-137.4) | 0.019 |
| AFP prior to LT (ng/mL) | 5.2 (1.1-17,718) | 4.7 (1.1-93.8) | 6.5 (1.4-17,718) | 0.003 |
| Number of HCC lesions | 2.0 (1.0-15.0) | 1.0 (1.0-3.0) | 4.0 (1.0-15.0) | <0.001 |
| >1 lesion | 105 (55.9%) | 48 (42.5%) | 57 (77.0%) | <0.001 |
| >2 lesions | 66 (35.1%) | 14 (12.4%) | 52 (70.3%) | <0.001 |
| >3 lesions | 45 (23.9%) | 0 (0%) | 45 (60.8%) | <0.001 |
| Largest lesion size (cm) | 3.2 (0.1-9.5) | 2.9 (0.1-8.0) | 3.5 (0.4-9.5) | 0.042 |
| Aggregate lesion size (cm) | 4.4 (0.1-27.5) | 3.5 (0.1-13.4) | 7.8 (0.4-27.5) | <0.001 |
| Microvascular invasion | 20 (10.6%) | 0 (0%) | 20 (27.0%) | <0.001 |
| Macrovascular invasion | 8 (4.3%) | 0 (0%) | 8 (10.8%) | <0.001 |
| Tumor differentiation |  |  |  |  |
| Poorly differentiated | 9 (4.8%) | 1 (0.9%) | 8 (10.8%) | 0.003 |
| Moderately differentiated | 98 (52.1%) | 53 (46.5%) | 45 (60.8%) | 0.055 |
| Well differentiated | 25 (13.3%) | 13 (11.4%) | 12 (16.2%) | 0.342 |
| Unknown | 56 (29.8%) | 47 (41.2%) | 9 (12.2%) | <0.001 |
| Incidental HCC | 12 (6.4%) | 9 (7.9%) | 3 (4.1%) | 0.371 |
| Post-LT AFP (ng/mL) | 3.0 (0.8-9,625) | 3.0 (0.8-9,625) | 3.2 (0.9-2,155) | 0.099 |
| Steroids >3 months after LT | 134 (71.3%) | 86 (75.4%) | 48 (64.9%) | 0.117 |
| Acute cellular rejection | 29 (15.4%) | 17 (14.9%) | 12 (16.2%) | 0.809 |
| Recurrence | 16 (8.5%) | 1 (0.9%) | 15 (20.3%) | <0.001 |
| *^1^* Median (range); n (%). ^2^ From LRT closest to the time of LT | | | | |
| *AFP, alpha-fetoprotein; HCC, hepatocellular carcinoma; LRT, locoregional therapy; LT, liver transplant; MASLD, metabolic associated steatotic liver disease; RFA, radiofrequency ablation; TACE, transarterial chemoembolization; TARE, transarterial radioembolization.* | | | | |
